# Diabetes Risk Assessment and Awareness in a University Academics and Employees

**DOI:** 10.14744/SEMB.2021.84770

**Published:** 2021-12-29

**Authors:** Tulin Yildiz, Senay Zuhur, Sayid Shafi Zuhur

**Affiliations:** 1. Tekirdag Namik Kemal University, Highschool for Health Sciences, Tekirdag, Turkey; 2. Department of Diabetes Education, Tekirdag Namik Kemal University Training and Research Hospital, Tekirdag; 3. Department of Endocrinology and Metabolism, Tekirdağ Namık Kemal University, Tekirdağ, Turkey

**Keywords:** Diabetes, FINDRISK, obesitye

## Abstract

**Objectives:**

Screening of the community for diabetes is generally costly and imposes a significant financial burden. Therefore, some non-invasive measures such as the Finnish Diabetes Risk Score (FINDRISK) Scale have been developed and are generally recommended for screening of people, particularly those with a high risk of diabetes. However, the screening of the university employees including academics with FINDRISK scale has not been performed so far. Therefore, in this study, we intended to assess the risk of diabetes by FINDRISC among the academics and other employees of a university as well as to make diabetes awareness among them.

**Methods:**

442 subjects were included in this study. “Diabetes awareness meetings” were organized, posters with awareness themes were displayed and brochures were distributed to academics and employees of our university. The FINDRISK was used for diabetes risk assessment. Participants’ height, weight, waist circumference, and body mass indexes were measured and were recorded.

**Results:**

The mean age of the participants was 36.76±9.05. About 62%, 67%, and 32% of the participants were females, married, and academic staff, respectively. The mean waist circumference and body mass index of the participants were 84.71±14.49 cm and 26.8±4.91 kg/m^2^, respectively, and the median FINDRISK score was 7 (3–10). The 10-year risk of developing diabetes, assessed by FINDRISK score was very high and high, moderate, mild, and low in 8, 10.6, 32.4, and 43.9% of the participants, respectively. Significant differences were found between FINDRISK scores according to gender, age, marital status, smoking status, and occupational positions of the participants (p<0.001 for all parameters). However, the FINDRISK scores of the academics were significantly higher than in other groups.

**Conclusion:**

Our study results suggest that the 10-year risk of developing diabetes is higher in academics compared to the other employees. Therefore, to raise awareness among people, diabetes prevention training is of paramount importance, regardless of the education levels of the people, to prevent or delay the development of diabetes.

Type 2 diabetes is a rapidly growing global health problem. The incidence of diabetes varies depending on age, gender, race, nutritional habits, genetic characteristics, and environmental factors.^[1,2]^

Turkey ranks third with diabetes frequency among European countries.^[3]^ In the Turkish Diabetes Epidemiology Study-I and -II performed 10 years apart, the prevalence of diabetes and prediabetes was found to be increased from 7.2% and 6.7% to 13.7% and 13.9%, respectively. On the other hand, 45% of patients with diabetes were not aware of their disease.^[4,5]^

Many of the risk factors associated with type-2 diabetes are preventable and manageable. The frequency of type-2 diabetes can be reduced or its occurrence can be delayed by taking and maintaining the necessary measures.^[6]^ Prevention of type-2 diabetes in healthy individuals is important not only for decreasing the risk of microvascular and macrovascular complications but also for improving the quality of life, decreasing the economic burden of the disease, and minimizing the physical, psychological, and social impact of the disease as well.

Although there is no global screening recommendation for type-2 diabetes, each country runs diabetes screening programs, taking into account their health indicators.^[7]^ The International Diabetes Federation (IDF) suggests that people at high risk for diabetes should first be identified using diabetes risk assessment tools such as the Finnish Diabetes Risk Score (FINDRISK) followed by biochemical analysis for diabetes.^[8]^ The FINDRISK has been developed as a result of a community-based cohort study in Finland in 2003 and is one of the detection tools that determine diabetes risk in adults by estimating the 10-year incidence of diabetes. FINDRISK scale consists of eight simple questions, including age (years), body mass index (BMI) (Kg/m^2^), physical activity (at least 30 min/day), daily vegetable-fruit consumption, family history of diabetes, high blood glucose value detected at any time, blood pressure and waist circumference measurement (cm), and has been translated into 15 different languages, and is used in community-based diabetes screening. FINDRISK has many advantages over other diabetes risk score measures for determining the risk of type-2 diabetes.^[9-11]^ The most important advantage of the FINDRISK is that it is a simple, noninvasive, and self-applicable tool in primary health-care facilities as well as screening for diabetes in the community. It reduces the number of people who need a laboratory test and consequently decreases the cost of screening.^[12]^ It is also a tool that can be understood by any health-care professional such as a diabetes education nurse, without any laboratory testing, and the risk score can be easily calculated.^[13-16]^

For the assessment of the risk of diabetes, several studies have been performed among different professions and social groups. However, the number of studies evaluating the risk of diabetes among university academics and employees, who are assumed to be an example of the general population with different levels of education, daily activity, and working conditions, is limited. Therefore, in this study, we intended to evaluate a university’s academics and employees for diabetes risk, to create training programs specific to this group, and to increase awareness of diabetes among them.

## Methods

This study conducted between September and November 2019 and included randomly selected 442 subjects among the academics and employees of the Tekirdag Namik Kemal University. To develop diabetes awareness among academics and employees, educational brochures describing diabetes, its risk factors, and complications were distributed. A poster exhibition was also prepared in all faculties of the university, demonstrating the definition of diabetes, its risk factors, and complications, followed by diabetes awareness meetings describing the same issues. At the end of the meeting, participants were evaluated by FINDRISK, to assess diabetes risk.

Anthropometric measurements of the participants were performed at the morning fasting state, using the height and weight measurements by researchers. The measurements were carried out with the subjects in light-weighted dresses, standing erect, bare-footed, and heels together. The BMI of the participants was calculated according to the formula: Weight in kilograms divided by height in meters squared. Subjects with a BMI of <18.50, between 18.5 and 24.9, between 2 and 29.9, and >30 were accepted as underweight, normal, overweight, and obese, respectively.

Waist circumference was measured at the end-expiration in a fasting state by taking the narrowest diameter between the arcus costarum and the processus Spina iliaca anterior superior, using an elastic tape measure. Following the World Health Organization obesity criteria, the normal waist circumference for female and male participants was accepted as 88 and 102 cm, respectively.

BMI measurement results and the results obtained from FINDRISK together with the Turkish Endocrinology and Metabolism Association guidelines for screening of diabetes were sent by e-mails to the participants.

Subjects <20-years-old, subjects with a previous diagnosis of any type of diabetes, subjects with a diagnosis of cognitive dysfunction, pregnant, subjects with a difficulty in hearing, or speech were not included in the study. The study was conducted following the declaration of Helsinki, and the study protocol was approved by the Tekirdag Namik Kemal University, Faculty of Medicine’s noninterventional ethics committee, and informed consent was obtained from all participants. The study received a grant from Tekirdag Namik Kemal University (NKUBAP.02.GA.19.215).

### FINDRISK

FINDRISK scale consists of eight simple questions, including age (years), BMI (Kg/m^2^), physical activity (at least 30 min/day), daily vegetable-fruit consumption, family history of diabetes, high blood glucose value detected at any time, blood pressure, and waist circumference measurement (cm). However, the questionnaire distributed to the participants was including eight questions included in the FINDRISK and six extra questions that may be directly or indirectly associated with diabetes including gender, professional position, marital status, smoking habit, and history of gestational diabetes, and polycystic ovary syndrome in female participants were added in the questionnaire.

The FINDRISK scale can be completed without any laboratory testing. The answer to each question is calculated with different weighted scores based on the risk increase associated with the relevant values in the regression model of the original cohort. The total score ranges from 0 to 26 as the sum of all scores from eight questions.^[10,16]^ With the risk assessment for diabetes in the next 10 years, participants with <7 points, between 7 and 11 points, between 12 and 14 points, between 15 and 20 points, and >20 points, were accepted as low risk (1%), mildly increased risk (4%), moderately increased risk (16%), high risk (33%), and very high risk (50%), respectively. After evaluation by the FINDRISK, participants were recommended to be screened for diabetes by laboratory analysis, and/or included in the diabetes prevention program as suggested by the Olgun et al. and Lindström and Tuomilehto.^[16,17]^

### Statistical Analysis

PASW Statistics 18 for Windows program was used for data input and statistical analysis. Mean±standard deviation and frequency were used to state results. The suitability of the data for normal distribution was evaluated with the Kolmogorov–Smirnov Test. Analysis of variance and independent sample T-Test was used for the analysis of parametric data, and the Mann–Whitney U and Kruskal–Wallis H tests were used for the analysis of non-parametric data. The relationship of continuous variables with each other was evaluated with the non-parametric Spearman’s correlation test. A p<0.05 was accepted as statistically significant.

## Results

A total of 442 subjects (274 female [62%] and 68 male [38%], mean age 36.76±9.05 years old [between 20 and 64 years old]), were included in this study. The baseline characteristics of the study participants are shown in Table 1, and the variables used for comparison according to the FINDRISK score are shown in Table 2.

**Table 1.** Baseline characteristics of the study participants (n=442)

**Variables**	**n (%)**
Gender	
Female	274 (62)
Male	168 (38)
Age	
<35	202 (45.7)
35–44	147 (33.3)
45–54	79 (17.9)
55–64	14 (3.1)
Marital status	
Married	296 (67)
Single	146 (33)
Professional position	
Academic staff	142 (32.1)
Administrative staff	167 (37.8)
Other	133 (30.1)
Smoking status	
Never smoked	221 (50)
Quit	72 (16.3)
Current smoker	149 (33.7)
Waist circumference (cm)	
Female (n=274)	
<80	176 (64.2)
80–88	43 (15.7)
>88	55 (20.1)
Male (n=168)
<94	70 (41.7)
94–102	56 (33.3)
>102	42 (25)
BMI (kg/m^2^)
<25	173 (39.1)
25–30	162 (36.7)
>30	107 (24.2)

BMI: Body mass index.

**Table 2.** FINDRISK score of the participants according to the variables used for the assessment of the FINDRISK

**Variables**	**(FINDRISK score points)**	**n (%)**
Age (Years)	<35 (0)	202 (45.7)
	35–44 (1)	147 (33.3)
	45–54 (2)	79 (17.9)
	55–64 (3)	14 (3.1)
	>64 (4)	-
Waist circumference female (cm)	<80 (0)	176 (64.2)
	80–88 (3)	43 (15.7)
	>88 (4)	55 (20.1)
Waist circumference male (cm)	<94 (0)	70 (41.7)
	94–102 (3)	56 (33.3)
	>102 (4)	42 (25)
BMI (kg/m^2^)	<25 (0)	173 (39.1)
	25–30 (1)	162 (36.7)
	>30 (3)	107 (24.2)
Exercising 30 min/day	Yes (0)	383 (86.7)
	No (2)	59 (13.3)
Frequency of consuming vegetables and fruits	Every day (0) Not every day (1)	235 (53.2) 207 (46.8)
History of using	No (0)	414 (93.7)
antihypertensive medications	Yes (2)	28 (6.3)
History of high blood glucose	No (0)	390 (88.2)
level at any time	Yes (5)	52 (11.8)
Family history of diabetes	No (0)	213 (48.2)
	Second-degree relative (3)	103 (23.3)
	A first-degree relative (5)	126 (28.5)
	Low (<7)	217 (49.1)
	Mild (7–11)	143 (32.4)
	Moderate (12–14)	47 (10.6)
	High (15–20)	33 (7.5)
	Very high (>20)	2 (0.5)

FINDRISK: Finnish diabetes risk score; BMI: Body mass index.

In the present study, mean waist circumference and BMI in female, male and all participants were 77.95±12.2 cm and 26.02±5.37 kg/m^2^, 95.73±10.67 cm and 28.11±3.75 kg/m^2^, and 84.71±14.49 cm and 26.81±4.92 kg/m^2^, respectively. However, as demonstrated in Table 3, BMI was significantly lower in females compared to males, in younger compared to older, in singles compared to married, in smokers compared to never smokers, and in other employees compared to administrative and academic staffs. On the other hand, the administrative staff had the highest BMI followed by academic staff. Among the participants, 219 (80%) of the female and 126 (75%) of the male participants had a waist circumference of <88 cm and 102 cm, respectively. However, only 173 (39%) of all participants had a BMI <25 Kg/m^2^, despite >85%, and 53% of participants reported a daily exercise of >30 min/day and regular consumption of fruits and vegetables. According to the total scores obtained from the FINDRISK scale, 0.5%, 7.5%, 10.6%, 32.4%, and 49.1% of the participants had very high, high, moderate, mild, and low risk, respectively. The association between age, gender, marital status, smoking habit, and profession with BMI is presented in Table 3. In our study, a significant difference was found in FINDRISK scores according to gender, age, BMI, marital status, smoking habits, and professional positions of the participants as well. As demonstrated in Table 4, FINDRİSK scores were significantly lower in females compared to males despite 12 (4.4%), and 39 (14.2%) of the female participants had a history of gestational diabetes and polycystic ovary syndrome. Nevertheless, FINDRISK scores were also significantly lower in younger compared to older, in marrieds compared to singles, in academic staffs compared to administrative staffs and other employees, in those who quit smoking compared to current smokers, and never smokers, and in those with high BMI as well. In this study, however, despite that the risk of diabetes was found to be increased by increasing BMI and age, according to the FINDRISK score, the risk of diabetes was the highest among the academic staff and the lowest among the other employees. In correlation analysis, a moderate linear correlation was found between FINDRISK scores and age and strong linear correlations were found between FINDRISK scores and BMI and waist circumference (r=0.427, p<0.001; r=0.692, p<0.001; and r=0.645 p<0.001, respectively).

**Table 3.** Comparison of BMI measurement results according to the descriptive features of the participants (n=442)

**Variables**	**n (%)**	**BMI (kg/m^2^)**	**p**
Gender			
Female	274 (62)	26.02±5.37	<0.001
Male	168 (38)	28.11±3.75	
Age			
<35	202 (45.7)	25.52±5.06	<0.001
35–44	147 (33.3)	27.17±4.32	
45–54	79 (17.9)	28.57±4.32	
55–64	14 (3.1)	31.69±5.59	
Marital status			
Married	296 (67)	27.46±4.64	<0.001
Single	146 (33)	25.49±5.2	
Smoking status			
Never smoked	221 (50)	26.08±5.02	0.005
Quit	72 (16.3)	27.49±4.43	
Current smoker	149 (33.7)	27.57±4.84	
Professional position			
Academical staff	142 (32.1)	26.96±4.85	<0.001
Administrative staff	167 (37.8)	27.63±4.8	
Other	133 (30.1)	25.62±4.91	

BMI: Body mass index.

**Table 4.** Comparison of FINDRISK score results According to the descriptive characteristics of the participants (n=442)

**Variables**	**n (%)**	**FINDRISK score**	**p**
Gender			
Female	274 (62)	6.64±4.63	0.034
Male	168 (38)	7.63±4.82	
Age			
<35	202 (45.7)	5.45±4.44	<0.001
35–44	147 (33.3)	7.44±4.34	
45–54	79 (17.9)	9.05±4.13	
55–64	14 (3.1)	13.64±4.94	
BMI (kg/m^2^)			
<25	173 (39.1)	3.61±2.98	<0.001
25–30	162 (36.7)	7.25±3.66	
>30	107 (24.2)	12.14±3.55	
Marital status			
Married	296 (67)	7.81±4.69	<0.001
Single	146 (33)	5.39±4.37	
Smoking status			
Never smoked	221 (50)	6.11±4.34	<0.001
Quit	72 (16.3)	8.6±5.02
Current smoker	149 (33.7)	7.58±4.85	
Professional position			
Academic staff	142 (32.1)	7.72±4.47	<0.001
Administrative staff	167 (37.8)	7.66±4.92	
Other	133 (30.1)	5.44±4.37	

FINDRISK: Finnish diabetes risk score; BMI: Body mass index.

## Discussion

In the present study, according to the FINDRISK score, 35 (8%), 47 (10.6%), 143 (32,4%), and 217 (49%) of the participants had very high, high, moderate, and low risk, for developing diabetes within 10 years, respectively. FINDRISK scores were also found to increase by increasing age BMI and waist circumference. On the other hand, a significant difference between the FINDRISK scores was found according to the gender, age, marital status, smoking status, and occupational positions of the participants. FINDRISK score was the highest among the academic staff and the lowest among the other employees, despite the BMI of the administrative staff was slightly higher compared to the academic staff.

The strong relationship between obesity and type-2 DM is well-known and people with a BMI of >25 kg/m^2^ have a higher risk of diabetes.^[18]^ According to the recent guidelines, hypertension, hyperlipidemia, impaired glucose tolerance, and signs of insulin resistance such as acanthosis nigricans, women with a history of polycystic ovarian syndrome (PCOS) or gestational diabetes, patients with cardiovascular disease, people with a sedentary lifestyle, and those who consume foods rich in saturated fat are also at high risk for developing type-2 DM. Type-2 diabetes can remain insidious for many years. During the period when the symptoms of the disease are not noticed, it is possible to delay or prevent the development of type-2 DM by intervening in the risk factors.^[19]^ It has been suggested that a change in lifestyle through diabetes prevention programs can reduce the incidence of type 2 diabetes by 58% in 3 years.^[20]^

Obesity, which is one of the most important risk factors for the development of type-2 DM, is a public health problem and its prevalence is steadily increasing around the globe. Although the frequency of obesity is higher among females; nowadays, it also increases rapidly among males.^[21]^ In our study, the waist circumference was higher than suggested in 20% of females and 25% of males. On the other hand, the mean BMI of all participants was 26.81±4.92 (between 17 and 46) and 60.9% of the participants had a BMI consistent with overweight and obesity. Although in correlation analysis, a strong linear correlation was found between waist circumference and BMI with FINDRISK scores (r=0.64, p<0.001 and r=0.69, p<0.001) and between waist circumference and BMI (r=0.836, p<0.001), the lower number of participants with a lower waist circumference compared to the number of participants with a lower BMI may be related to a higher number of female participants, particularly young female participants. Our study results are in line with those reported previously. In the study of Kulak et al., the waist circumferences were found to be above the suggested values in 68.2% of males and 72.4% of females, and most of them were overweight and obese according to the BMI values.^[22]^ Therefore, to prevent obesity and diabetes, lifestyle interventions should be recommended by providing weight control, healthy nutrition rich in vegetables and unsaturated fat, and increasing daily physical activity. Therefore, planning awareness-raising activities by health-care professionals in society, even in well-educated people like academics, is important in this regard.

Studies suggested a lower frequency of diabetes in females compared to males.^[23]^ According to a report of the IDF published in 2017, 8.4% of females (203.9 million) and 9.1% of males (221 million) aged between 20 and 79-years-old had diabetes.^[23]^ Nevertheless, the proportion of females and males with diabetes was predicted to increase to 9.7% and 10% by 2045, respectively.^[23]^ In our study, although the median FINDRISK score was 7 (3–10), male participants had higher FINDRISK scores compared to females (7.63±4.82 vs. 6.64±4.63, p=0.034). This result of our study was in line with that reported by Cevik et al.^[24]^ In their study, although statistically non-significant, the FINDRISK scores were higher in males compared to female participants (11.99±6.21 vs. 12.67±7.01, p>0.05). On the other hand, in another study conducted by Kulak et al., the mean FINDRISK score was higher in females compared to male participants.^[22]^ Some other studies performed in participants with different professions also demonstrated higher FINDRISK scores in females compared to males.^[24-27]^ While the FINDRISK scores were not significantly different between males and females in some other studies.^[26,28,29]^ We think that the lower FINDRISK scores in female compared to male participants found in our study may be due to the inclusion of the actively working females without a sedentary lifestyle, instead of housewives that were included in some other studies.

The incidence of obesity has increased dramatically and exceeds 50% and 30% among the 45–74 and 45–64 age groups of women and men, respectively.^[13]^ Although the frequency of obesity is higher in females, in recent years, a trend toward a rapid increase in obesity is observed among males as well.^[13]^ Our study results confirm that trend and suggest a higher BMI in male compared to female participants which were consistent with FINDRISK scores between both genders. Therefore, according to our study results, males, particularly those working in the bureau, have a higher risk of diabetes. Hence, diabetes awareness programs should particularly focus on this subgroup of people.

BMI increases with an increase in waist circumference. A unit increase in BMI value increases the risk of developing type 2 diabetes by 25%.^[30]^ In our study, a strong positive correlation was found between FINDRISK scores with BMI and waist circumference (r=0.69, p<0.001 and r=0.64, p<0.001). These results of our study are consistent with prior studies suggesting a strong association between FINDRISK scores with BMI and waist circumference.^[22,24,31]^ Therefore, the risk of diabetes is increased with an increase in waist circumference as well as BMI. Therefore, it is important to emphasize the relationship between weight loss, particularly the fat around the abdominal area, and diabetes, and to raise awareness about the ideal waist circumference in the community.

One of the modifiable risk factors in the development of type 2 diabetes is physical inactivity. The benefits of moderate physical activity for at least 150 min/week for controlling weight gain and preventing type 2 diabetes are well-known.^[13]^ In our study, 13.3% of the participants were found to be physically inactive. We think that the participants of our study were aware of the health benefits of exercise on general health conditions, perhaps due to their high education levels of the participants. For instance, in a study conducted by Kulak et al., the physical inactivity among the participants was 59%.^[22]^ This outcome of our study suggests that the education level of the people may have a direct impact on health outcomes. Therefore, suggestions for increasing physical activity among people are of paramount importance. It should be aimed to establish institutional policies aimed at promoting physical activity in the workplace and to create areas for physical activity for the employees of institutions.

Healthy eating habits and the regular consumption of vegetables and fruits with high fiber are also important for the prevention of type-2 diabetes.^[13]^ In our study, 46.8% of the participants were not regularly consuming fiber-rich vegetables and fruits. In the study of Kulak et al., 39.8% of the participants were not consuming fiber-rich vegetables and fruits, which is close to our study results.^[22]^ The results of our study also suggest that most people, including those with higher education levels, do not care about the health benefits of healthy feeding. The absence of consumption of fiber-rich vegetables and fruits in our study participants, which is one of the modifiable risk factors for the development of diabetes, could also have increased the diabetes risk score in our study participants. Therefore, in diabetes prevention programs, practical solutions, and the transformation of individual eating habits into healthy eating habits should be carefully emphasized.

Having a family history of diabetes is also one of the non-modifiable risk factors for the development of type-2 diabetes. In our study, a first-degree family history of diabetes was present in 28.5 of the participants. However, the 10-year mild, moderate, and high risk of development of diabetes determined by FINDRISK in our study was roughly 50%. Therefore, we could say that modifiable risk factors of diabetes are as important as the genetic background of the people.

Along with age, which is another non-modifiable risk factor, weight gain increases the risk of diabetes. In our study, a moderate linear correlation was found between age and BMI with the FINDRISK score of the participants (r=0.365, p<0.001, r=0.427, p<0.001). In a study by Kulak et al., 30% and 38.7% of participants aged 45–54 and 38.7% and 55–64 had a higher risk of diabetes and they also found a significant relationship between FINDRISK score and age.^[22]^ Cevik et al. also demonstrated a significant increase in the frequency of diabetes with an increase in age and weight.^[24]^ Hence, the middle age and older people should be the target of a diabetes prevention program to decrease the risk of diabetes in the community.

In our study, a significant difference was found between the marital status of the participants with the BMI and FINDRISK scores. Married participants had significantly higher BMI and FINDRISK scores as compared to single participants. This situation may be explained as married couples may affect each other’s eating habits and lifestyle, and lifestyle factors such as eating habits may contribute to the higher BMI and FINDRISK scores in married subjects. Aksu also demonstrated a significant difference between the marital status of the participants and the risk of diabetes.^[32]^ However, while there was a significant relationship between smoking status and higher diabetes risk in our study, no significant relationship between smoking and higher diabetes risk was observed in studies conducted by Aksu (2018) and Viitasalo et al.^[32,33]^ Although there is not sufficient data in terms of the relationship between smoking habit and the development of type 2 diabetes, smoking is known to increase the risk of atherosclerosis in individuals with and without diabetes.^[34]^ However, this result of our study should be confirmed in further studies.

In our study, a significant difference was found between a professional position with weight and diabetes risk. Academic and administrative staff had higher BMI and FINDRISK scores as compared to other employees (Tables 3 and 4). We believe that academic and administrative staff may be more sedentary during the day and consequently that condition may affect their weight and may increase diabetes risk among them. However, there are no data to demonstrate the diabetes risk among academics. Occupational positions and working environments are some of the important factors affecting health status. Therefore, solutions to increase physical activity and healthy nutrition during working hours should be found to decrease the diabetes risk among the academic and administrative staff of the universities.

PCOS and a history of diabetes during pregnancy are independent risk factors for the development of type 2 diabetes.^[35]^ PCOS is a common endocrine disease affecting between 8-18% of women of reproductive age.^[36]^ More than 2% of these women may develop diabetes each year, so screening for diabetes is important at regular intervals in patients with PCOS.^[37]^ However, in the present study, no significant relationship was found between the previous history of PCOS and the risk of diabetes determined by the FINDRISK score among female participants. The reason for the non-significant risk of diabetes in women with a history of PCOS in our study can be explained by the fact that PCOS was present in a small proportion of women. In a study by Lisa et al., women with a history of PCOS had a higher diabetes risk score.^[38]^ They conclude that their waist circumference and BMI values may increase their diabetes risk score. On the other hand, a significant difference was found between the FINDRISK scores among the female participants who had a history of diabetes in pregnancy and those who had not. The relationship between diabetes during pregnancy and the development of postpartum diabetes is a well-known condition.^[35]^ Therefore, in diabetes prevention programs, it should be emphasized that women with a history of PCOS and pregnancy have a higher risk for the development of diabetes, and screening at appropriate intervals should be performed to diagnose diabetes or prediabetes at the earlier stages.

## Conclusion

Our study results show that the risk of diabetes increases by increasing age and waist circumference. Smoking status, marital status, and occupational positions of the participants were other factors associated with a higher risk of diabetes assessed by the FINDRISK score. Academics had the highest diabetes risk among the participants of our study. Therefore, education about diabetes prevention in individuals with a high risk of diabetes to raise awareness among people, irrespective of their education level, is important to prevent or delay the development of diabetes.

### Disclosures

**Ethics Committee Approval:** The study was conducted following the declaration of Helsinki, and the study protocol was approved by the Tekirdag Namik Kemal University, Faculty of Medicine’s noninterventional ethics committee, and informed consent was obtained from all participants. The study received a grant from Tekirdag Namik Kemal University (NKUBAP.02.GA.19.215). (2018.176.12.10).

**Peer-review:** Externally peer-reviewed.

**Conflict of Interest:** None declared.

**Authorship Contributions:** Concept – T.Y.; Design – T.Y.; Supervision – S.S.Z.; Materials – S.S.Z.; Data collection &/or processing – S.S.Z.; Analysis and/or interpretation – S.S.Z.; Literature search – T.Y.; Writing – T.Y. , S.S.Z.; Critical review – S.S.Z.
